# Traumatic Brain Injury With Concomitant Myocardial Infarction: A Clinical Dilemma

**DOI:** 10.7759/cureus.50898

**Published:** 2023-12-21

**Authors:** Nour Abukalam, Resshme Kannan Sudha, Muneer Al Marzooqi

**Affiliations:** 1 Emergency Medicine, Tawam Hospital, Al Ain, ARE

**Keywords:** acute st elevation myocardial infarction, multidisciplinary teams, intracranial hemorrhage, altered level of consciousness, fall from height, major trauma, severe head injury

## Abstract

A 51-year-old male patient was brought to the emergency department (ED) by paramedics after an unwitnessed fall from a height while he was working. He sustained a severe head injury with a low Glasgow Coma Scale (GCS). After securing his airway and stabilizing the patient, a CT scan of the brain was done that revealed bilateral subdural hematomas, and an electrocardiogram (EKG) revealed an ST elevation inferior wall myocardial infarction (MI), which was suggested to be the cause of his fall. With the presence of two concomitant life-threatening medical conditions, it was a predicament which of the two pathologies to target first in treatment. Ultimately, a management plan was decided following a multidisciplinary urgent meeting in the ED, which was attended by all respective teams. Initial conservative management with close neurological and cardiovascular monitoring in the intensive care unit (ICU) was deemed the safest option in this case.

## Introduction

Either traumatic brain injury (TBI) or acute coronary syndromes (ACS) on their own are common entities that are present in the emergency department (ED) [1,2]. However, this case brought an unusual occurrence of both conditions simultaneously and put the medical team in a dilemma as to which is the best order to treat the patient and reverse the pathologies as they were both time-sensitive. Emphasizing that both conditions are critical and that the management of one may affect the other, this case will highlight our approach and the importance of quick multidisciplinary meeting input in managing mixed and complicated cases.

## Case presentation

Emergency medical services (EMS) brought a 51-year-old male to the resuscitation area, immobilized on a backboard and with a cervical collar applied following an unwitnessed fall from height. The patient was said to be working on a ladder around 3-5 m high before falling down, where he was then found on the floor with an altered level of consciousness. On arrival to the ED, initial vital signs showed a blood pressure of 161/97 mmHg, heart rate of 150 beats/minute, respiratory rate of 20 breaths/minute, and temperature of 36.8°C, and he was maintaining oxygen saturation at 100% on room air. The patient was agitated with a Glasgow Coma Scale (GCS) of 11/15. The only apparent signs of injury were noted on the head (a hematoma over the right parietal lobe). Given the patient's low GCS, past medical history was difficult to obtain. Moreover, the circumstances of his fall could not be determined as it was an unwitnessed event. The patient had a witnessed seizure in the ED, which was aborted with an intravenous (IV) benzodiazepine, and then, he underwent rapid sequence intubation (RSI) for better control and protection of the airway as his GCS dropped to 6. The patient was then sent for urgent CT imaging, and further workup was done upon his return to the resuscitation area, including blood tests and a bedside electrocardiogram (EKG).

**Figure 1:**
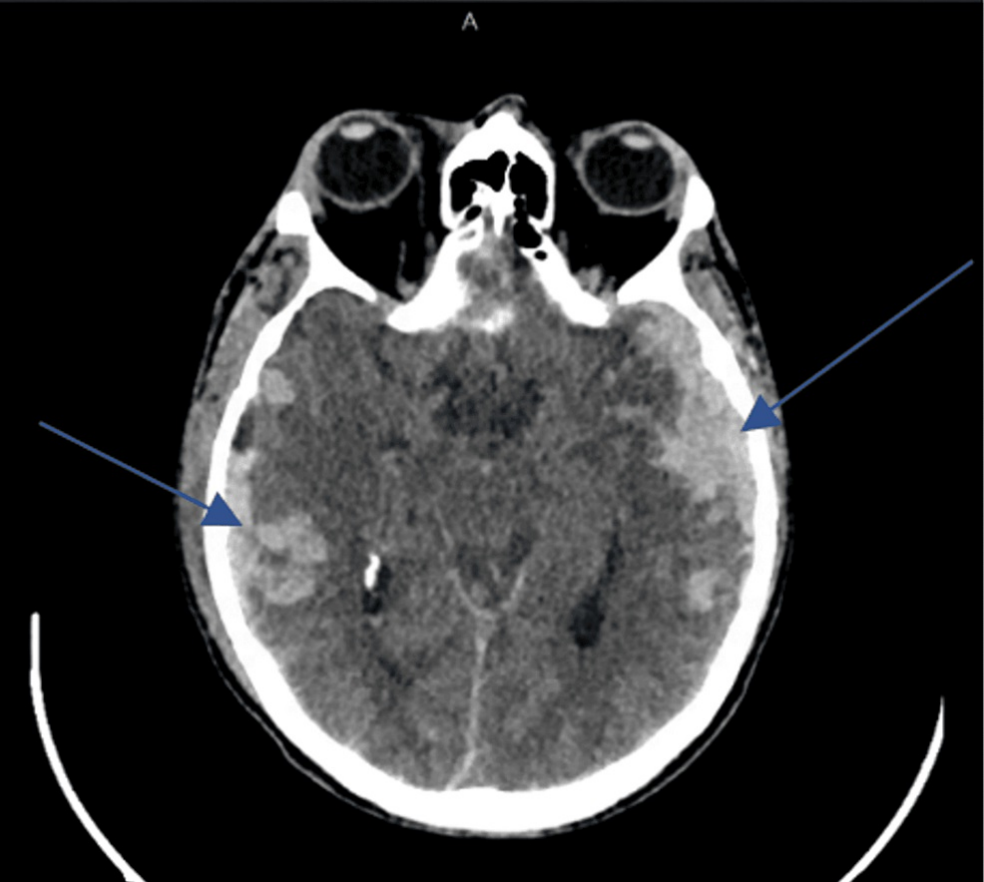
Head CT demonstrating bilateral subdural hematoma in the right and left temporal lobes (blue arrows)

**Figure 2:**
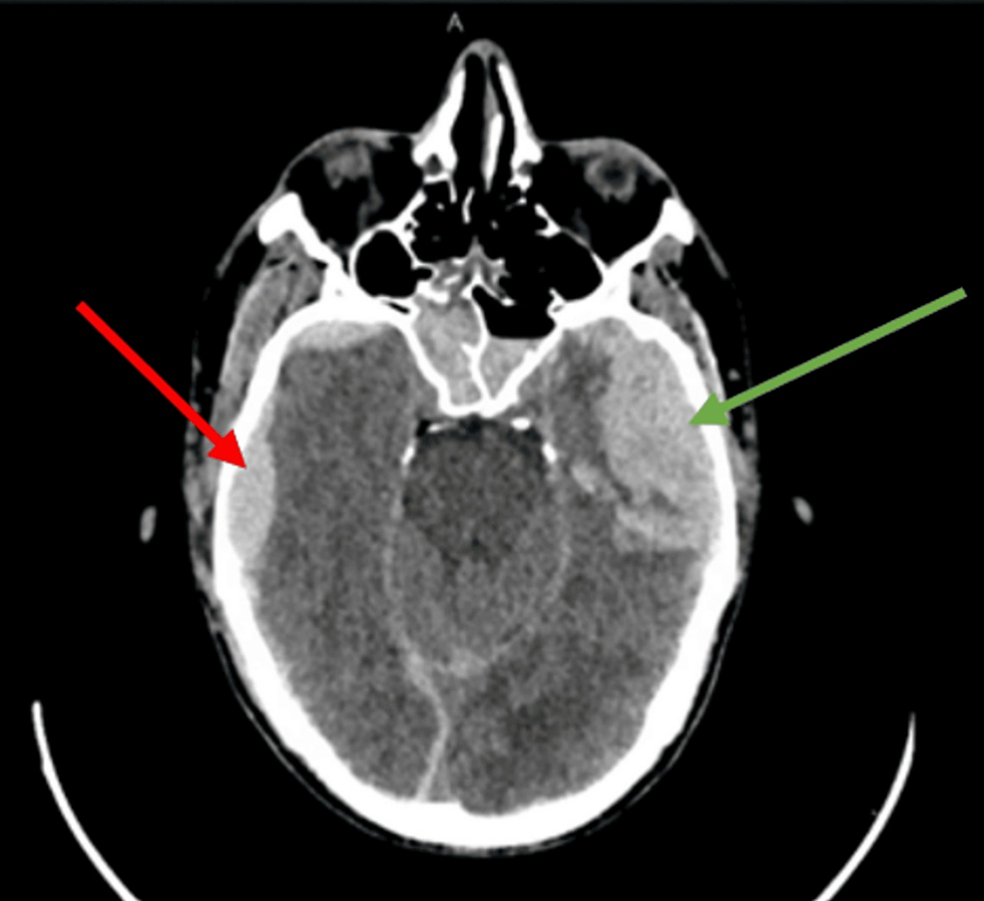
Head CT demonstrating right-sided epidural hematoma (red arrow) and left subdural hematoma (green arrow)

The head CT (CT of the brain {CTB}) revealed multiple bony fractures with minimal displacement and showed bilateral subdural hematomas, one on the left temporal lobe measuring 50 × 23 mm and another on the right temporal lobe with a negligible size. Also, a right epidural hematoma around 9.5 mm was noted in the right temporal lobe. There were areas of thick contusion more conspicuous in the left frontal and both parietal lobes (Figures 1, 2). No definite signs of tonsillar herniation were seen. At that time, there was no midline shift, as shown in the attached image.

**Figure 3:**
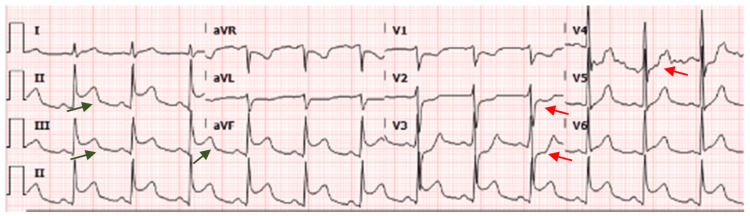
EKG on day 1 demonstrating ST elevation in inferior leads (green arrows) with reciprocal changes in anteroseptal leads (red arrows) EKG: electrocardiogram

An EKG done post imaging showed an ST elevation inferior myocardial infarction (MI) (Figure 3). Blood investigations sent showed a prothrombin time (PT) of 11.6 seconds, international normalized ratio (INR) of 1.12, D-dimer of 35.20 mg/L, hemoglobin of 12.2 g/dL, and troponin T of 103 ng/L. All remaining laboratory results were within normal limits.

In addition to TBI as a primary differential diagnosis, a number of differential diagnoses were considered as potential causes for the fall. These included and were not limited to mechanical fall, equipment failure, stroke, syncope, seizure, hypoglycemia, vertigo, electrocution, ACS, and arrhythmias.

CTB revealed bilateral subdural hematoma, subarachnoid hemorrhage, and a right epidural hematoma with a basilar skull and petrous bone fracture. The EKG revealed evidence of ST elevation inferior wall MI. Given the scenario and course of events, it was imperative to come up with a definitive management plan.

The clinical dilemma was prioritizing treatment in a patient with two potentially life-threatening diagnoses requiring interventional cardiology for emergent percutaneous coronary intervention (PCI), neurosurgery for the evacuation of hematomas, and intensive care unit (ICU) for admission.

Our decision was to quickly conduct a multidisciplinary team meeting in the ED with interventional cardiology, neurosurgery, and critical care. The challenge in managing the ST elevation myocardial infarction (STEMI) was that the patient required loading with anticoagulants and antiplatelets as per local and international protocols and guidelines before being shifted to the catheterization laboratory in order to ensure the best chances of recanalization and patency of the blocked coronary artery. However, this ultimately could have worsened his intracranial bleeding and led to deleterious effects. Given that the patient was hemodynamically and clinically stable post endotracheal intubation, conservative management with watchful waiting and close neurological observation using an intracranial pressure (ICP) monitor and cardiovascular observation was decided to be done in the ICU. The use of titrated IV fluids and vasopressors was advised if the patient became hypotensive during the course of admission. It was also advised that a pacemaker be inserted in case he developed bradycardia or atrioventricular blocks. As per the interventional cardiologist, coronary intervention would have probably caused more harm than good. Moreover, the neurosurgical team advised for the insertion of an ICP monitor and intracranial pressure-lowering measures, but no acute surgical intervention or decompression was required in the first few hours of care. It was advised to repeat a follow-up CTB after six hours to look for the expansion of the hematoma or signs of herniation.

Serial CTB scans repeated after six hours, on day 1 and day 2, did not show progression of the brain hematomas. However, CTB on day 3 showed mild worsening of brain contusions but no signs of midline shift or herniation. Regular intracranial pressure (ICP)-reducing measures were adopted during the ICU admission course. A follow-up CTB done on day 9 was suggestive of a midline shift and signs of impending uncal herniation. As a result, the patient was taken for a left-sided decompressive craniectomy, which was uneventful.

**Figure 4:**
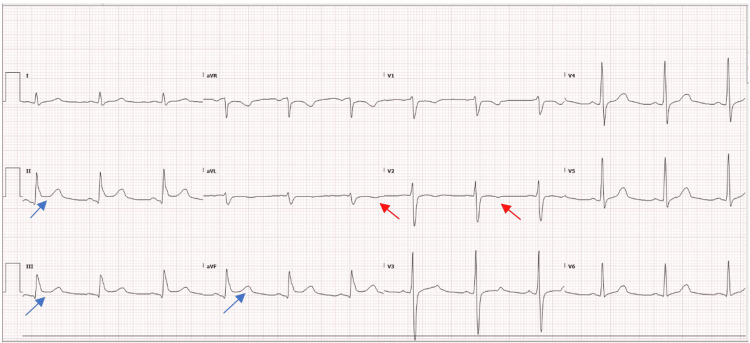
EKG on day 2 demonstrating ST elevation in inferior leads (blue arrows) with T wave inversions in aVL and V2 (red arrows) EKG, electrocardiogram; aVL, augmented vector left

**Figure 5:**
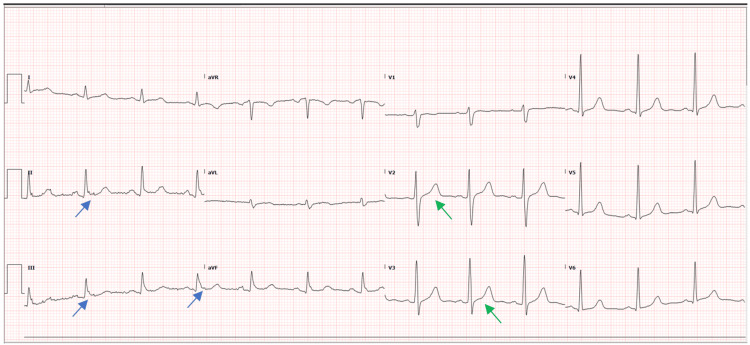
EKG on day 14 demonstrating resolution of ST elevation in the inferior leads (blue arrows) and T wave inversions in anteroseptal leads (green arrows) EKG: electrocardiogram

In addition, he was followed by the cardiology team over the course of his ICU stay. Serial EKGs done showed ST elevation inferior myocardial Infarction on the first and second days of admission with subsequent EKGs showing the resolution and reversal of the STEMI changes (Figures 4, 5). Furthermore, serial troponin levels showed a downward trend (60 ng/L, 47.3 ng/L, and 33.2 ng/L on day 2, day 3, and day 8, respectively). Formal echocardiography did not reveal any marked regional wall motion abnormalities. He was not placed on antiplatelets due to the risk of worsening intracranial hemorrhage.

The patient responded well to the initial conservative management and subsequent decompressive craniectomy and showed gradual clinical recovery. He was weaned off the ventilator and was extubated successfully on day 13 of admission and maintained a GCS of 10/15.

On day 14 of admission, his GCS score had improved to 12/15. He partially responded to the examination instructions and was found to have right-sided hemiparesis. The patient was then transferred to a neurological rehabilitation facility. After four months of neurological rehabilitation, the patient returned to our hospital for left-sided cranioplasty to replace the bone flap. His GCS score was 15/15, and the right-sided hemiparesis improved. The patient recovered well after the procedure and was discharged on postoperative day 5. Overall, the patient clinically improved and was able to resume daily activities with support.

## Discussion

The simultaneous occurrence of TBI and ST elevation on EKG poses a challenging clinical scenario, demanding a careful evaluation of the interplay between neurological and cardiovascular insults. It could not be established whether the patient in this case had acute MI or acute intracerebral hemorrhage first, as he presented to the ED with both conditions concomitantly. This case report delves into the complexities inherent in managing a patient with concomitant TBI and EKG changes, shedding light on the diagnostic and therapeutic dilemmas encountered in such cases.

The presence of ST elevation on EKG in the context of TBI suggests a potential intersection between the neurological and cardiac systems. TBI, especially in its severe forms, is often accompanied by an acute stress response and sympathetic overdrive. This surge in sympathetic activity may contribute to myocardial ischemia and catecholamine-mediated myocardial stunning, thereby manifesting as ST elevation on EKG. Other EKG changes include QT prolongation and T wave inversion [3,4]. Understanding this neuro-cardiac axis becomes crucial for navigating the diagnostic and therapeutic challenges posed by these concurrent pathologies.

The diagnostic landscape is significantly complicated when confronted with both TBI and ST elevation on EKG. The altered mental status and neurological deficits associated with TBI may mask or obscure the typical symptoms of myocardial infarction. Additionally, interpreting EKG changes in the presence of neurological injury presents a unique challenge. Distinguishing whether the ST elevation is due to primary cardiac pathology or a consequence of sympathetic activation secondary to TBI requires a nuanced diagnostic approach. Satria and Gumilang [5] and Heo et al. [6] highlight the importance of ruling in a cerebral cause in patients demonstrating ST elevation in their EKG prior to the initiation of antiplatelet and anticoagulant agents in their case reports describing a case of intracranial hemorrhage mimicking ST elevation MI and subarachnoid hemorrhage misdiagnosed as an acute ST elevation MI, respectively. In Hashemian et al.'s study of elderly patients more than 50 years of age, individuals experiencing intracranial hemorrhage demonstrated notably elevated occurrences of associated myocardial infarction compared to those with different forms of brain hemorrhage. The presence of ST-segment elevation was identified as having a positive predictive value of 71.4% in males and 25% in females for accurately diagnosing a true myocardial infarction linked to a hemorrhagic brain event [7].

Coagulation abnormalities commonly accompany traumatic injuries, exhibiting a progression from a hypercoagulable to a hypocoagulable state. In cases of coagulopathy induced by TBI, D-dimer is rapidly identified shortly after the injury, whereas prothrombin time and partial thromboplastin time are recognized later, reaching their highest levels 3-6 hours after the TBI. Plasma D-dimer levels have a positive correlation with trauma severity and, hence, are predictive of a poor prognosis. Fortunately, in this case, although the D-dimer level was elevated, the patient had a good prognosis [8-10].

The therapeutic challenges in this scenario are manifold, encompassing both the acute and long-term phases of care. The administration of antithrombotic and antiplatelet agents for MI management may raise concerns about exacerbating intracranial hemorrhage or compromising neurological outcomes. While the yearly incidence of intracranial hemorrhage among patients on dual antiplatelet therapy is 0.2%-0.3%, the total number of intracranial hemorrhage cases linked to antiplatelet therapy is not negligible [11]. The study conducted by Cao et al. highlights that the decision to treat acute MI or acute intracerebral hemorrhage first depends on the patient's hemodynamic stability. Cardiac catheterization may be performed without antithrombotic or antiplatelet use in cases of hemodynamic instability or STEMI after acute intracerebral hemorrhage [12]. Patlolla et al. reported that most patients with acute MI with intracranial hemorrhage less often required coronary angiography, percutaneous coronary intervention, or coronary artery bypass grafting as compared to those without intracranial hemorrhage [13].

Patients aged ≥85 years with STEMI were more likely to be managed conservatively. Patients who underwent invasive strategies had lower mortality rates than those managed conservatively, as per Yudi et al. [14], Lee et al. [15], and Bangalore et al. [16]. Our patient did not undergo any invasive coronary intervention and did not undergo any blood thinning to prevent the risk of bleeding. Hence, neurological observations, cardiac monitoring, and vital stabilization were performed. Striking a delicate balance between preventing further cardiac events and mitigating neurological complications becomes a critical decision point, requiring close collaboration between cardiology and neurocritical care teams. There is little evidence regarding the management principles of concomitant acute myocardial infarction and intracerebral hemorrhage.

The resolution of this clinical dilemma hinges on effective multidisciplinary collaboration. The coordination between cardiology, neurology, trauma, and critical care specialists is paramount in navigating the intricate challenges posed by concomitant TBI and MI. Timely communication and shared decision-making become integral components of a patient-centered approach, ensuring that interventions in one domain do not inadvertently exacerbate the challenges in the other.

The confluence of TBI and MI introduces uncertainties in predicting both neurological and cardiovascular outcomes. According to Cao et al., 50% of patients with concomitant acute STEMI and acute intracerebral hemorrhage have poor prognosis [12]. Prognostication becomes challenging, and the long-term impact on the patient's quality of life requires careful consideration. This inherent uncertainty reinforces the importance of ongoing reassessment and adaptive management strategies. Fortunately, in this patient's case, the patient recovered well and had a good prognosis.

## Conclusions

It is important to thoroughly investigate a patient presenting with trauma and look beyond the distracting injuries. Our patient in this case presented with a decreased level of consciousness, and an EKG revealed an incidental STEMI. Consider conducting immediate multidisciplinary team meetings involving critical care, neurosurgery, and cardiology in cases presenting with concomitant acute MI and acute intracerebral hemorrhage. Multidisciplinary involvement is vital in the management of complicated cases with concomitant pathology occurring simultaneously.
